# *The Plant Genome* special section: Modern improvement of tropical crops

**DOI:** 10.1002/tpg2.20482

**Published:** 2024-06-30

**Authors:** Stella Salvo, John Derera

**Affiliations:** ^1^ Bayer Crop Science Chesterfield Missouri USA; ^2^ CGIAR International Institute of Tropical Agriculture Ibadan Nigeria

In 2021, global food insecurity affected over 2 billion people, highlighting the urgent need for sustainable solutions (FAO et al., 2022). Climate change exacerbates these challenges, impacting farmers worldwide and compromising healthy diets (Ortiz‐Bobea et al., 2021). To address this, low‐ to middle‐income countries (LMICs) require increased crop productivity across diverse species (van Zonneveld et al., 2023).

This special issue offers insights into research for development, focusing on plant breeding and genetics, providing methods and tools to combat the devastating effects of climate change. Showcasing cutting‐edge genomic technologies applied to tropical crop breeding emphasizes the critical role of genetic resources in strengthening public breeding programs. Additionally, a review of tropical crops such as bananas underscores the importance of modernizing tools in the public sector. Highlighting advancements in transformation systems and genome editing which can be promising scalable technologies for LMICs.

This issue provides practical insights into estimating genetic gain and modernizing breeding programs for crops like maize and cowpea, utilizing digital tools and modeling for effective decision‐making in research. The strength of this special issue lies in its broad coverage of crop species. It outlines steps needed to integrate genetic information into breeding programs of varying maturity levels. From understanding genetic diversity to employing marker‐assisted breeding and genomic selection, these articles illustrate significant progress in modernizing tropical crop breeding programs. Facilitated by collaborations, they offer inspiration and pave the way for further innovation, emphasizing the potential of plant genomics in enhancing food security worldwide.
